# Author Correction: Causal inference from cross-sectional earth system data with geographical convergent cross mapping

**DOI:** 10.1038/s41467-023-43055-y

**Published:** 2023-11-10

**Authors:** Bingbo Gao, Jianyu Yang, Ziyue Chen, George Sugihara, Manchun Li, Alfred Stein, Mei-Po Kwan, Jinfeng Wang

**Affiliations:** 1 College of Land Science and Technology, China Agricultural University, Beijing, China; 2 Key Laboratory of Remote Sensing of Agricultural Disasters, Ministry of Agriculture and Rural Affairs, Beijing, China; 3 Faculty of Geographical Sciences, Beijing Normal University, Beijing, China; 4 Scripps Institution of Oceanography, University of California, San Diego, La Jolla, CA USA; 5 School of Geography and Ocean Science, Nanjing University, Nanjing, China; 6 Faculty of Geo-Information Science and Earth Observation (ITC), University of Twente, Enschede, The Netherlands; 7 Department of Geography and Resource Management, and Institute of Space and Earth Information Science, The Chinese University of Hong Kong, Hong Kong, China; 8 Department of Human Geography and Spatial Planning, Utrecht University, Utrecht, the Netherlands; 9 State Key Laboratory of Resources and Environmental Information Systems, Institute of Geographic Sciences and Nature Resources Research, Chinese Academy of Sciences, Beijing, China

**Keywords:** Environmental impact, Environmental impact, Social sciences

Correction to: *Nature Communications* 10.1038/s41467-023-41619-6, published online 21 September 2023

The original version of this Article contained an error in the results, which incorrectly read:

‘The zero *ρ* of **temperature** xmap farmland NPP indicate that farmland NPP is not a cause of **temperature**. And the much-smaller *ρ* of **precipitation** xmap farmland NPP majorly result from the above-introduced enslaved effect from the strong causal influence of **precipitation** on farmland NPP. In other words, farmland NPP can partially reflect **precipitation**.’

The correct version now reads:

‘The zero *ρ* of **precipitation** xmap farmland NPP indicate that farmland NPP is not a cause of **precipitation**. And the much-smaller *ρ* of **temperature** xmap farmland NPP majorly result from the above-introduced enslaved effect from the strong causal influence of **temperature** on farmland NPP. In other words, farmland NPP can partially reflect **temperature**.’

This has been corrected in both the PDF and HTML versions of the Article.

