# Supplementary material for: Causal inference from cross-sectional earth system data with geographical convergent cross mapping
Source: Nat Commun. 2023 Sep 21;14:5875. doi: 10.1038/s41467-023-41619-6 (PMC10514035; doi:10.1038/s41467-023-41619-6)
Supplement: Supplementary file 1 — Supplementary Information [file 41467_2023_41619_MOESM1_ESM.pdf]

Supplementary Materials for

Causal Inference from cross-sectional Earth System data with Geographical Convergent Cross Mapping

Bingbo Gao[1,2#] , Jianyu Yang[1,2#], Ziyue Chen[3*], George Sugihara[4], Manchun Li[5], Alfred Stein[6], Mei-Po Kwan[7,8], Jinfeng Wang[9*]

[1] *College of Land Science and Technology, China Agricultural University, Beijing 100193, China.*

[2] *Key Laboratory of Remote Sensing of Agricultural Disasters, Ministry of agriculture and rural affairs, Beijing 100083, China.*

[3] *Faculty of Geographical Sciences, Beijing Normal University, Beijing 100875, China.*

[4] *Scripps Institution of Oceanography, University of California, San Diego, 9500 Gilman Drive, La Jolla, CA 92093, USA.*

[5] *School of Geography and Ocean Science, Nanjing University, Nanjing 210023, China.*

[6] *Faculty of Geo-Information Science and Earth Observation (ITC), University of Twente, Hengelosestraat 99, 7514 AE Enschede, The Netherlands.*

[7] *Department of Geography and Resource Management, and Institute of Space and Earth Information Science, The Chinese University of Hong Kong, Hong Kong, China.*

[8] *Department of Human Geography and Spatial Planning, Utrecht University, 3584 CB Utrecht, the Netherlands.*

[9] *State Key Laboratory of Resources and Environmental Information Systems, Institute of Geographic Sciences and Nature Resources Research, Chinese Academy of Sciences, A11 Datun Road, Beijing 100101, China.*

[#] *These authors contributed equally to this work.*

Corresponding author.

Email: Ziyue Chen (zychen@bnu.edu.cn) or Jinfeng Wang (wangjf@lreis.ac.cn)

Section S1: Overview of causal inference models

In Table S1, we summarized the mainstream causal inference methods. According to their target application fields, we firstly divide them into two general categories, one for stochastic system and the other for dynamic system. The most important difference between them is that, the former assumes random distribution and separable relationships, while the latter assumes deterministic trends and intertwined relationships. The structural causal modeling framework and potential outcome framework are two main branches of causal inference methods for stochastic system, and the prediction-based Granger causation also belongs to the stochastic system[1]. While the methods based on state space reconstruction[2] are specially for the dynamic systems. We will introduce the structural causal modeling framework, potential outcome framework, Granger causation and state space reconstruction in brief as follows. For more details, please refer to the related works we cited in the table S1.

The structural causal modeling framework was proposed from Wright [3] and developed by Pearl [4, 5]. It emphasizes the use of casual graph, which is directed acyclic, with the node representing variables and edges representing casual relationships. It includes two major category of methods. The first category aiming at causal-effect estimation which requires casual graphs known beforehand. The other category is causal-network learning, which aims to discover casual associations from observation data[6]. For the causal effect estimation category, Do calculus, Front-door adjustment and Back-door adjustment, can be used for the manipulation of the causal graph built beforehand, while information prevention, variable randomization and statistical adjustment can be adopted to estimate the causal effect[5, 7]. It can take full advantage of prior-knowledge to estimate casual effects. For the causal-network learning category, PC model, named after the inventors Peter and Clark, is a basic method, which builds a complete undirected graph first and then iteratively deletes the spurious edges based on the conditional independence[8]. Its main assumption includes causal sufficiency, Markov condition and faithfulness. The causal sufficiency requires that all common cause variables are included. The Markov condition relates the probability distribution P of variables with the structure of causal graph G, by requiring that given its parents, a variable (corresponding to a node in the graph) should be conditionally independent from other variables (excluding its parents and descendants). Faithfulness in further require that every conditional independence relation in P is entailed by the Markov condition applied to G (i.e. a one to one map). FCI, RFCI and CCD are improved versions of PC algorithm[9, 10]. They can automatically discover potential casual associations from big data. But when employed for spatial causation inference, they

may generate spurious Markov equivalent graphs. PCMCI improves PC by employing an additional refinement based on momentary conditional independence (MCI) and the assumption that cause precedes effect[10]. PCMCI has an excellent performance in identifying causal associations, yet specified for time series data, rather than spatial cross-sectional data. To eliminate spurious Markov equivalent graphs, additional assumptions are set to recognize the causal direction, for such models as Additive Noise Model (ANM)[11], Linear Non-Gaussian Acyclic Model (LiNGAM)[12], and Nonlinear Gaussian additive noise models (nonlinear Gaussian ANMs)[11]. When applied to spatial cross-sectional data, they only work in specific cases satisfying assumptions on the function and noise.

The potential outcome framework was proposed by Neyman and Fisher, and developed by Rubin[13, 14]. It attempts to imitate the randomized controlled trials (RCT) by adjusting the variables. It can be divided into two categories, regular assignment mechanisms and regular assignment mechanisms with noncompliance[15]. In addition to the stable unit treatment value assumption (SUTVA), regular assignment mechanisms assume that the assignment mechanism is individualistic, probabilistic, and unconfounded, while regular assignment mechanisms with noncompliance relax the unconfoundedness assumption by allowing one-sided noncompliance and two-sided noncompliance[13, 16]. The SUTVA assumes that (1) the potential outcomes of different spatial units do not interfere with each other, and (2) all treatment levels are included so that the outcome should remain unchanged if the same treatment is assigned. The individualistic assumption is that the assignment probability of the treatment to one unit is independent from the covariates and potential outcomes for other units; the probabilistic assumption limits the assignment probability to numbers between zero and one; and the unconfoundedness assumption requires the assignment probability to be independent from the outcomes. Regular assignment mechanisms adopt imputation, weighting, blocking, and matching to adjust variables to imitate the designed randomized experiments, while regular assignment mechanisms with noncompliance often adopt instrumental variables to eliminate the confoundings. Regular assignment mechanisms can obtain unbiased estimation of the treatment effect when assumptions are satisfied, and regular assignment mechanisms with noncompliance estimate the treatment effect with an unobserved confounder by regarding the instrumental variable as a secondary treatment. The spatial difference in difference (SDID)[17] and spatial regression discontinuity (SRD)[18] are specially designed for spatial inference. The assumption of SDID is the common trend (units in treatment group and control group have the same trend) and unconfoundness, while SRD assumes that the spatial discontinuity boundary exists and individuals close to the boundary are similar. SRD takes spatial cross-sectional data as input and can identify the local treatment effect, while SDID requires

observations in two stages, before and after the treatment, and cannot manage spillover effects. Potential outcome framework can estimate the effect of a treatment, yet cannot discover casual associations.

Granger causation test is established based on the difference of prediction precision of the effect variable with or without the joining of a cause variable. Its assumption includes temporal precedence(cause precedes effect), information completeness (all common cause and effect variables should be included), temporal invariance (casual associations remains constant over time) and separability (information of cause variables is not contained in the effect variable) [18, 19]. It requires time series data as input and cannot be applied to spatial cross-sectional data.

For dynamic systems, the state space reconstruction theory has been adopted to identify and measure the casual associations[2]. CCM[20], CMS[21], PCM[22] are presentative methods for time series data, among which CCM can reliably detect the direction and strength of causal associations in weak-to-moderate coupling systems, is most frequently used. However, causal inference methods for spatial cross-sectional data in dynamic systems are absent. Therefore, GCCM was developed in this study to extract causal associations from spatial cross-sectional data and estimating the corresponding causal effects.

Table S1. Overview of mainstream causal inference methods

Systems	Framework	Category	Models	Major Assumptions	Input	Advantages	Limitations
Stochastic systems	Structural causal modeling framework[6]	Causal effect estimation[5, 7]	Do calculus, Front-door adjustment, Back-door adjustment, information prevention, variable randomization and statistical adjustment[5, 7]	Causal graph is known	Causal graph, Time series or spatial cross-sectional observation data	Can take full advantages of human knowledge	Causal associations is required beforehand
		Causal network learning[8]	PC, FCI, RFCI, CCD[9, 10]	Causal Sufficiency, Markov Condition, Faithfulness	Time series or spatial cross-sectional observation data	Can automatically discover potential casual associations from big data	Cause spurious Markov equivalent graphs
			PCMCI[10]	Causal Sufficiency, Markov Condition, Faithfulness, cause precede result	Time series observation data	Can Eliminate spurious Markov equivalent graphs, and have advantageous detection power	Cannot be used to cross-sectional data
			Additive Noise Model (ANM)[11]	Causal Sufficiency, Markov Condition, Faithfulness, the noise is additive	Time series or spatial cross-sectional observation data	Can identify causal graph for most functions and noise distributions	Cause spurious Markov equivalent graphs
			Linear Non-Gaussian Acyclic Model (LiNGAM)[12]	Causal Sufficiency, Markov Condition, Faithfulness, functions are linear and independent noise follows a non-Gaussian distribution	Time series or spatial cross-sectional observation data	Can identify correct causal graph for linear functions with non-Gaussian noise	Works in specific cases, fails with unmeasured confounders

		Causal network learning	Nonlinear Gaussian additive noise models (Nonlinear Gaussian ANMs) [11]	Causal Sufficiency, Markov Condition, Faithfulness, functions are nonlinear and three times differentiable, the noise follows a Gaussian distribution.	Time series or spatial cross-sectional observation data	Can identify correct causal graph for nonlinear and three times differentiable functions with Gaussian noise	Works in specific cases, fails with unmeasured confounders, intransitive.
Stochastic systems	Potential outcome framework[15]	Regular assignment mechanisms[12]	Imputation, weighting, blocking, and matching[12]	Stable unit treatment value assumption (SUTVA), the assignment mechanism is individualistic, probabilistic, and unconfounded	Time series or spatial cross-sectional observation data	Can obtain unbiased estimation of the treatment effect	Cannot discover casual associations
			Spatial difference in difference[17]	Common trend, unconfoundness	Paired spatiotemporal observation data	Can manage spillover effects which violate SUTVA	Cannot discover casual associations
			Spatial regression discontinuity[18]	Spatial discontinuity boundary exist and individuals close to the boundary are similar	Spatial observation data	Can identify of the local treatment effect	Cannot discover casual associations
		Regular assignment mechanisms with noncompliance[12]	Instrumental variables for with One-Sided Noncompliance[12]	Stable unit treatment value assumption (SUTVA), the assignment mechanism is individualistic, probabilistic, and one-sided noncompliance	Instrumental Variables, Temporal or spatial observation data	Can estimate the treatment effect with an unobserved confounder	Cannot discover casual associations

Stochastic systems	Potential outcome framework		Instrumental variables for with Two-Sided Noncompliance[12]	Stable unit treatment value assumption (SUTVA),The assignment mechanism is individualistic, probabilistic, and two-sided noncompliance	Instrumental Variables ,Temporal or spatial observation data	Can estimate the treatment effect with an unobserved confounder	Cannot discover casual associations
	Prediction based		Granger test[15]	Temporal precedence, information completeness, temporal invariance and separability	Time series observation data	Can identify casual links and directions between random variable	Cannot be used to cross-sectional data
Dynamic systems	State space reconstruction[2]	Temporal	CCM[20], CMS[21], PCM[22]	Deterministic dynamic systems	Time series observation data	Can reliably detect the direction and strength of causal associations in weak-to-moderate coupling systems	Cannot be applied to cross-sectional data
		Spatial	GCCM	Deterministic dynamic systems	Spatial cross-sectional observation data	Can reliably detect causal associations in weak-to-moderate coupling systems or leading causation direction in strong coupling systems	Specified for cross-sectional data

Section S2: soil pollution case

In this case, The soil pollution data were obtained from the National Geochemical Survey database of the U.S. covering Illinois and Indiana (https://mrdata.usgs.gov/geochem/)[23]. The soil heavy metals and pollution source data are stored as raster data, with 126 by 132 pixels and the spatial resolution as 5000 meters.

a. Soil Cd concentration

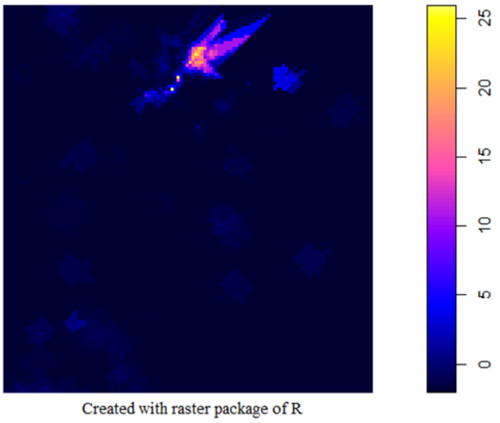

b. Soil Mg concentration

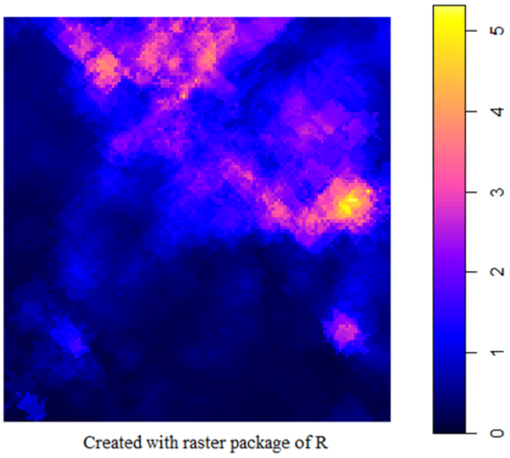

c. Soil Pb concentration

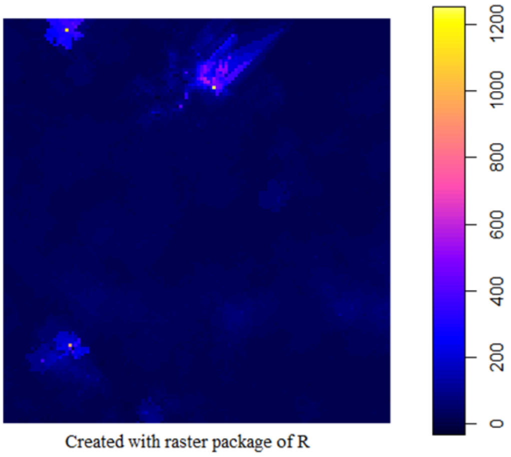

Fig. S1 Maps of soil heavy metals

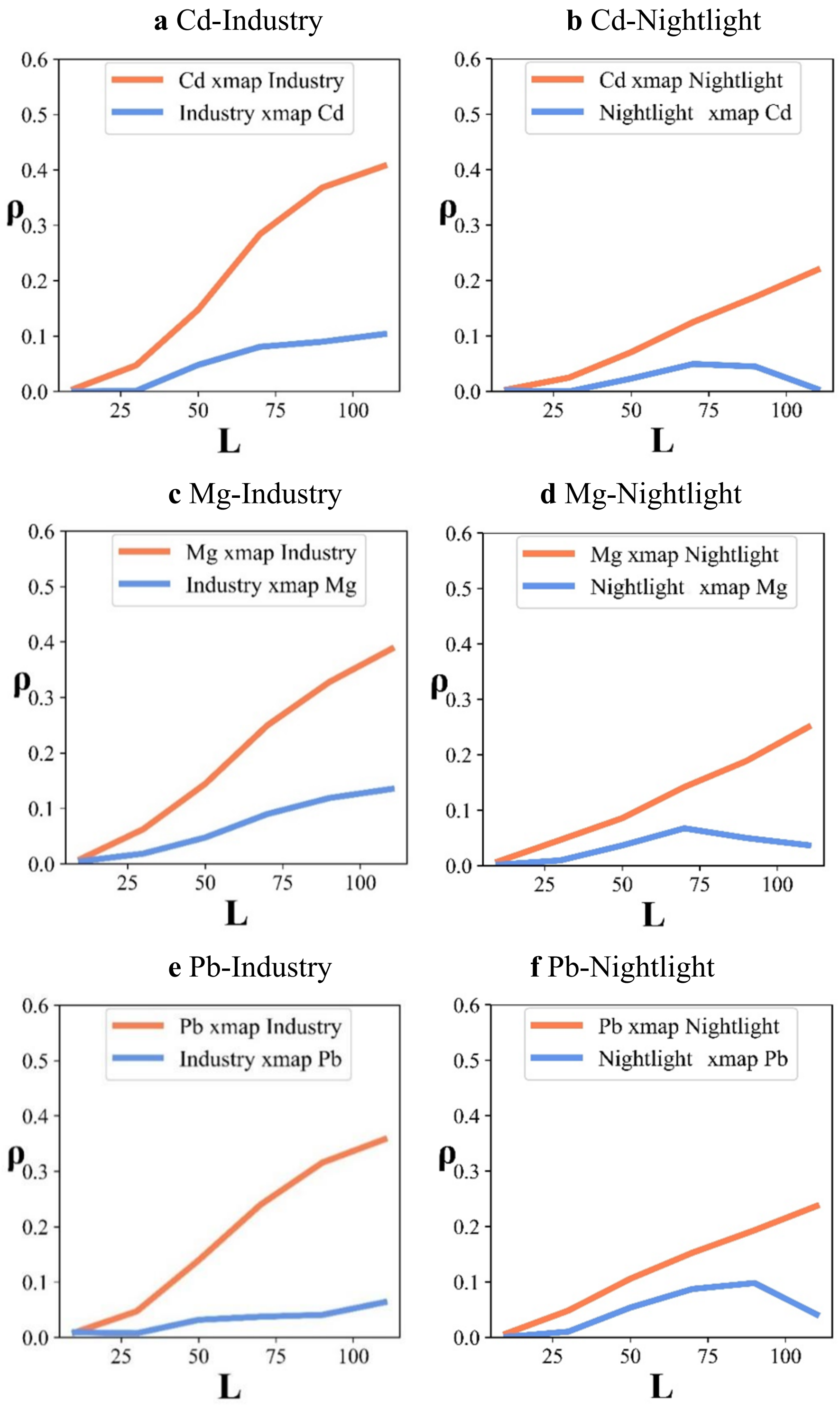

Fig. S2 Cross mapping prediction of soil heavy metals with GCCM.

a and **b** are the cross-mapping prediction results of Cd, **c** and **d** are the cross-mapping prediction results of Mg, **e** and **f** are the cross-mapping prediction results of Pb.

Table S2. Causation inference results for soil heavy metals (HM) and influencing factors

Method	Factor	Cu		Cd		Mg		Pb	
		r/ρ/*b*	*p/ste*	*r*/ρ/*b*	*p/ste*	*r*/ρ/*b*	*p/ste*	*r*/ρ/*b*	*p/ste*
Pearson Correlation	Industry	-0.08	0.05	-0.07	0.08	-0.07	0.07	-0.07	0.10
	Nightlight	-0.03	0.45	-0.03	0.43	-0.04	0.04*	-0.04	0.28
GCCM	Industry xmap HM	0.05 (-0.03,0.13)	0.09	0.1 (0.03,0.18)	0.00**	0.14 (0.06,0.21)	0.00**	0.06 (-0.01,0.14)	0.05
	HM xmap Industry	0.37 (0.3,0.43)	0.00**	0.41 (0.34,0.47)	0.00**	0.39 (0.32,0.45)	0.00**	0.36 (0.29,0.42)	0.00**
	Nightlight xmap HM	0.01 (-0.07,0.08)	0.44	0 (-0.07,0.08)	0.46	0.04 (-0.04,0.11)	0.17	0.04 (-0.04,0.12)	0.15
	HM xmap Nightlight	0.25 (0.18,0.32)	0.00**	0.22 (0.14,0.29)	0.00**	0.25 (0.18,0.32)	0.00**	0.24 (0.16,0.31)	0.00**
LiNGAM	HM→Industry	0.00	0.04	0.00	0.04	0.00	0.04	0.00	0.04
	Industry→HM	0.00	11.77	0.00	0.82	0.00	0.55	0.00	28.43
	HM→Nightlight	0.00	13.56	0.00	13.38	0.00	13.33	0.00	13.56
	Nightlight→HM	0.00	12.34	0.00	0.88	0.00	0.59	0.00	29.26

**** correlation is significant at the 0.01 level, * correlation is significant at the 0.05 level.**

***r*/ρ/*b* *r* is for Pearson correlation, ρ is for GCCM, *b* is for LiNGAM**

***p/ste p* is is significance p-value for Pearson correlation and GCCM, *stde* is the standard deviations of the estimated residuals of LiNGAM**

xmap means cross-mapping prediction, HM means heavy metals,→means cause; (*,*) is the 95% confidence interval.

Section S3: Population density case

In this case, the population density data of China were obtained from the National Bureau of Statistics of China, and the climate and topographical data were obtained from the data set published in Earth System Science Data (https://essd.copernicus.org/articles/11/1931/2019/). In order to generate the spatial neighboring matrix, we deleted the isolated counties before analysis.

The population density and influencing environmental factors are displayed in Fig. S3. As there exists an obvious linear trend from southeast to northwest in both the population density and environmental factors, we removed the linear trend separately using linear regression[24, 25]. In each linear regression, the coordinates of the center of county were used as independent variables, and the population density or environmental factor were taken as dependent variables. By subtracting the fitted values from the population density and influencing factors respectively, their residues and corresponding polygons were taken as the input of GCCM.

a Population density

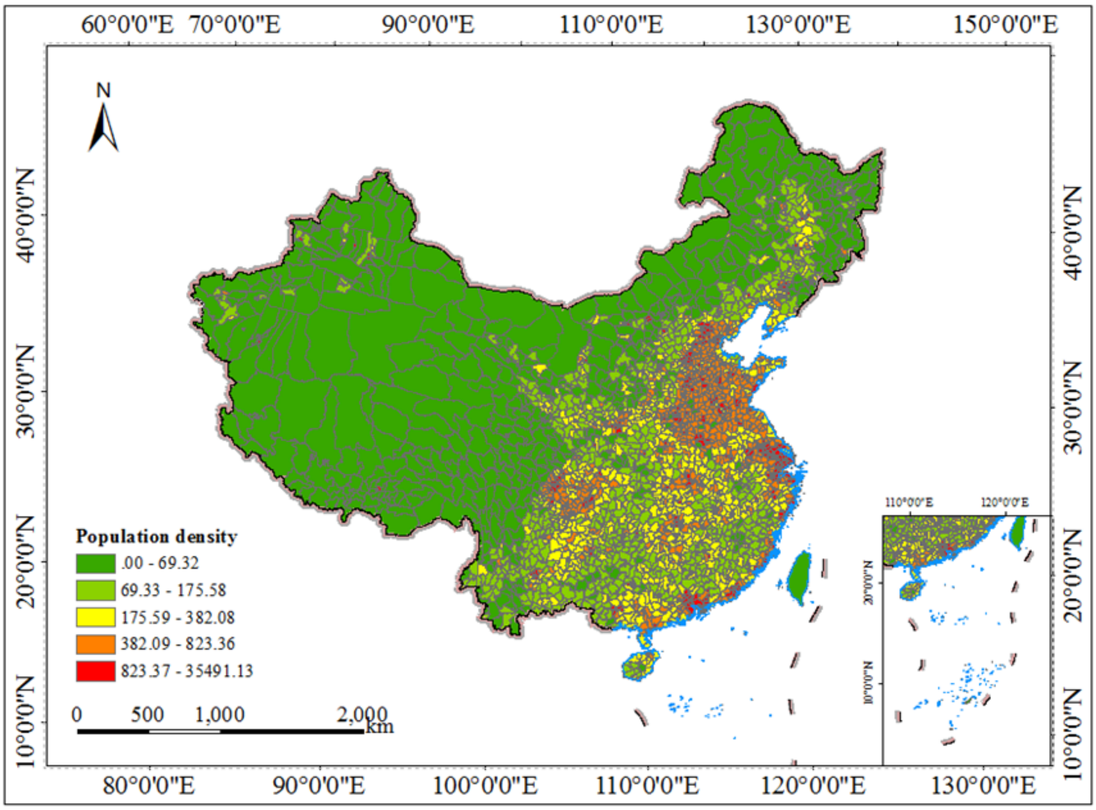

b Elevation

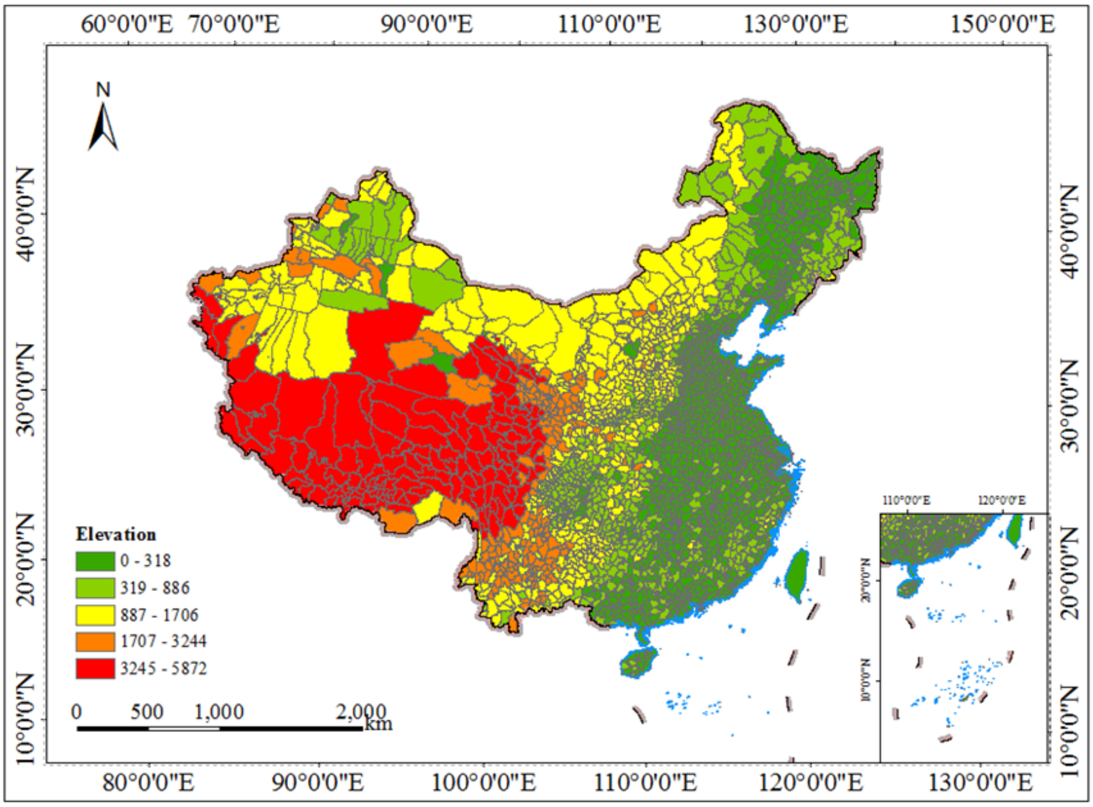

c Precipitation

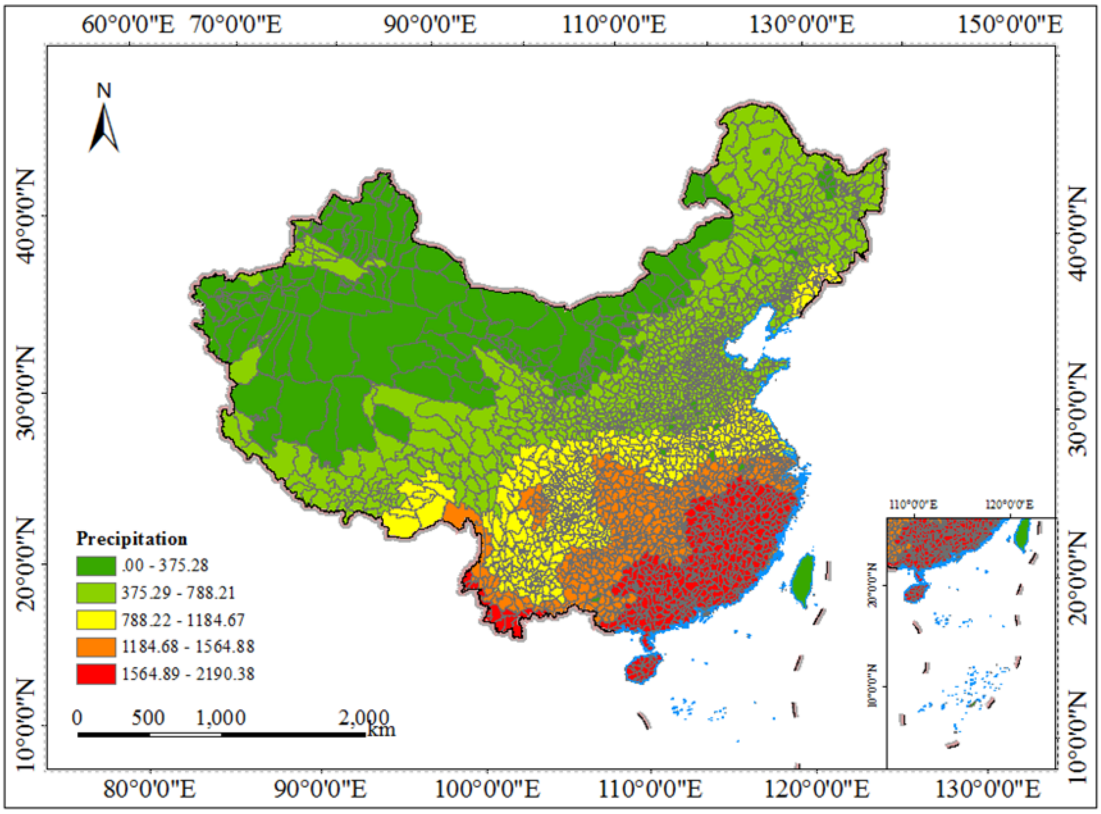

d Slope

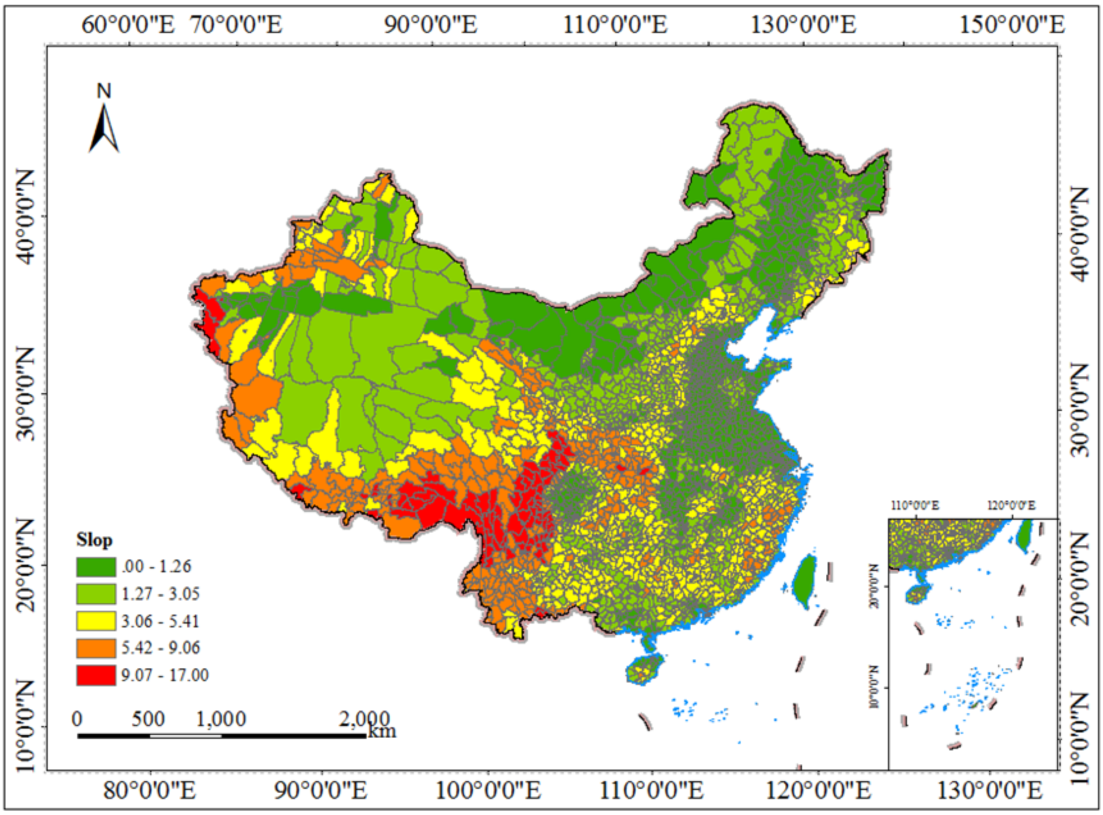

e Temperature

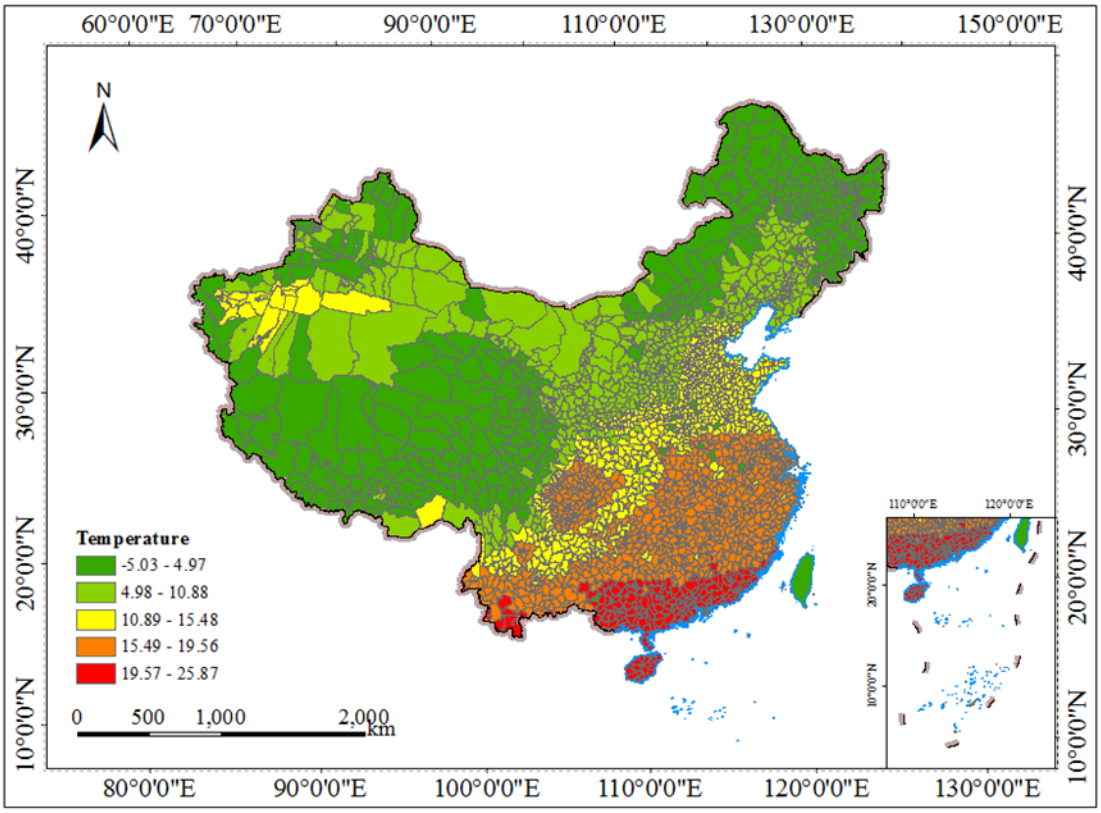

Fig. S3 Population density and influencing environmental factors of China. a Population density, **b** Elevation, **c** Precipitation, **d** Slope

Table S3. Causation inference results for population density and influencing environmental factors

Method	Relationship	Precipitation		Temperature		Elevation		Slop	
		r/ρ/b	*p/ste*	*r/ρ/b*	*p/ste*	*r/ρ/b*	*p/ste*	*r/ρ/b*	*p/ste*
Pearson Correlation		0.06	0.00**	0.15	0.00**	-0.18	0.00**	-0.23	0.00**
GCCM	Envs xmap Population	0.27 (0.23,0.3)	0.00**	0.31 (0.28,0.35)	0.00**	0.36 (0.33,0.39)	0.00**	0.26 (0.22,0.29)	0.00**
	Population xmap Envs	0.64 (0.62,0.66)	0.00**	0.63 (0.61,0.65)	0.00**	0.64 (0.61,0.66)	0.00**	0.48 (0.45,0.51)	0.00**
LiNGAM	Population → Envs	0.00	371.01	0.00	4.92	0.00	758.46	0.00	2.16
	Envs → Population	0.00	2601.02	0.00	2572.48	-1.08	2559.19	-364.03	2522.61

**** correlation is significant at the 0.01 level, * correlation is significant at the 0.05 level.**

r/ρ/b r **is for Pearson correlation,** ***ρ*** **is for GCCM,** ***b*** **is for LiNGAM**

p/ste p **is is significance** ***p*****-value for Pearson correlation and GCCM,** ***stde*** **is the standard deviations of the estimated residuals of LiNGAM**

xmap means cross-mapping prediction, Envs means environmental factors,→means cause, (*,*) is the 95% confidence interval

Section S4: Farmland NPP case

In this case, the NPP data were MOD17A3V055 downloaded from http://files.ntsg.umt.edu/data/NTSG_Products/MOD17/MOD17, with the spatial resolution as 1 km. The precipitation and temperature data were obtained from the data set published in Earth System Science Data with the url as https://essd.copernicus.org/articles/11/1931/2019/. We first calculated the average NPP, precipitation and temperature from 2000 to 2015 to get the spatial cross-sectional data of China, then masked them with farmland of China, and finally transformed the resolution from 1km to 10 km. The farmland data were obtained from the from the China Multi-period Land Use Land Cover Remote Sensing Monitoring Data Set (CNLUCC)[26]. The land use type of four periods (2000, 2005, 2010 and 2015) were employed to only retain the pixels of stable farmlands, which kept unchanged in all four periods.

The averaged farmland NPP, precipitation and temperature of China are displayed in Fig.S4. There also exists an obvious linear trend from southeast to northwest in both the population density and climate factors. So we removed the linear trend separately using the linear regression[20, 21]. In each linear regression, the coordinates of each grid were used as independent variables, and the farmland NPP or climate factor were taken as dependent variables. By subtracting the fitted values from the farmland NPP and influencing factors respectively, their residues as well as corresponding spatial grids were taken as the input of GCCM.

a Farmland NPP

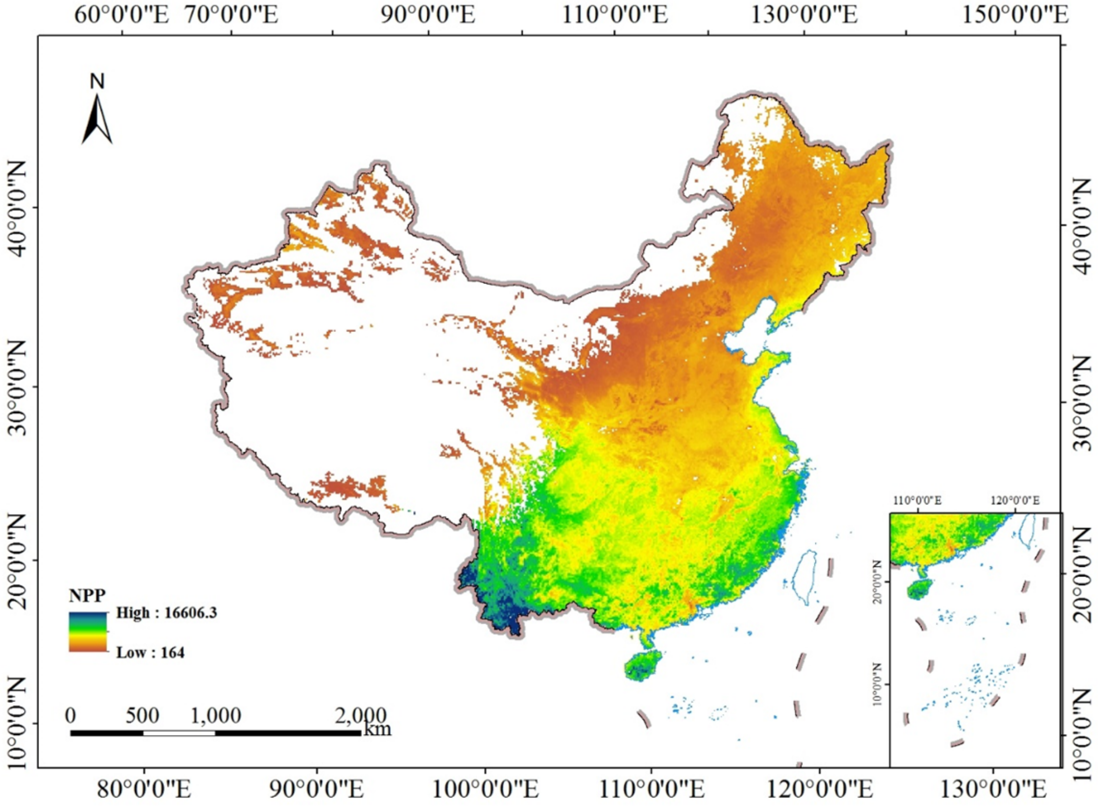

b Farmland Precipitation

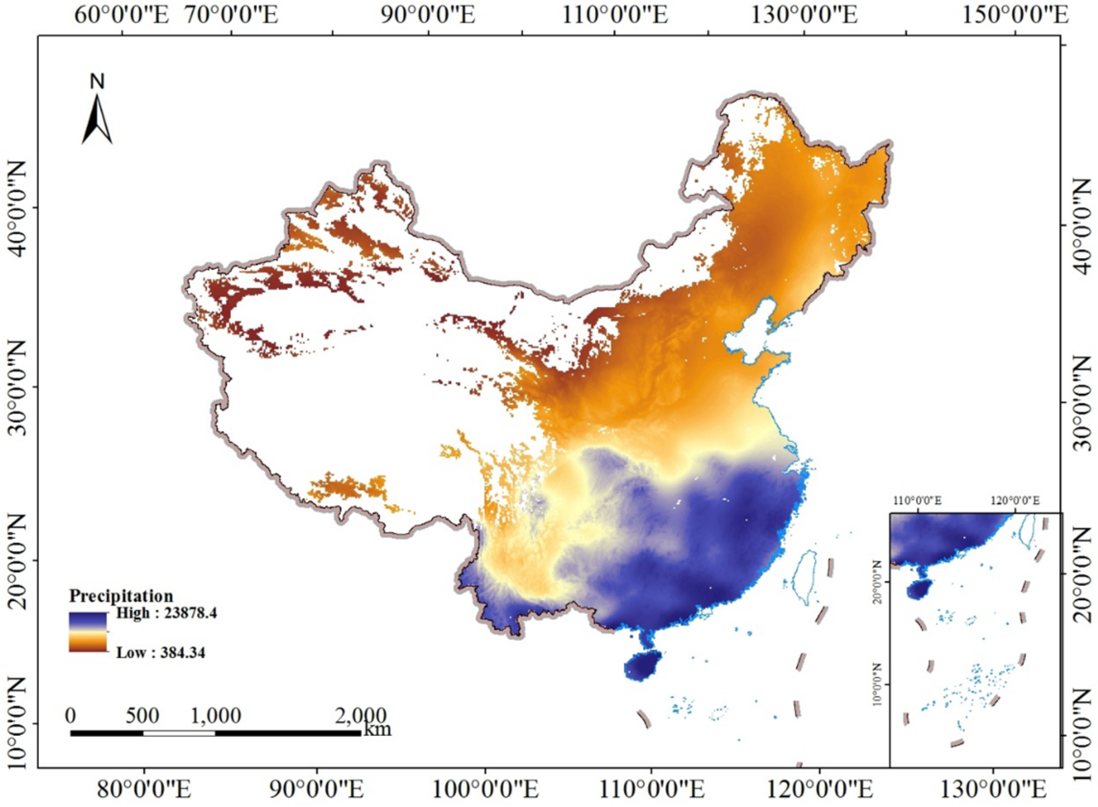

c Farmland Temperature

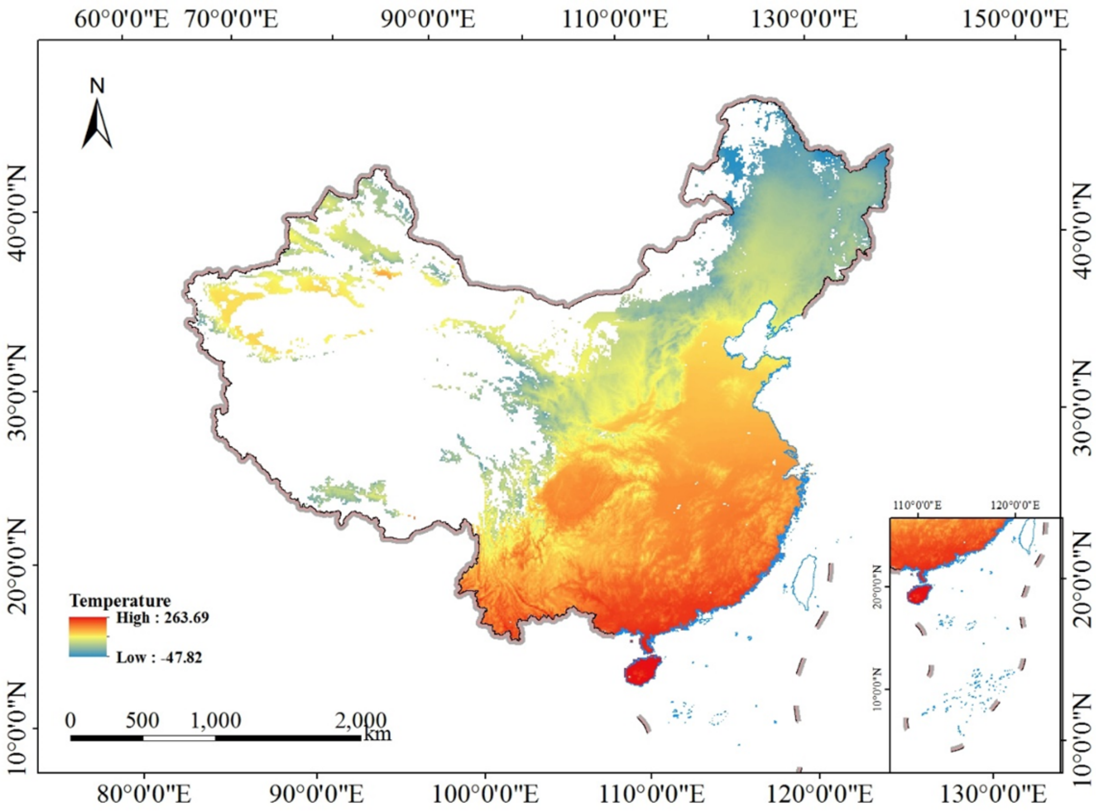

Fig. S4 Farmland NPP and influencing climate factors of China. a Farmland NPP, **b** Farmland Precipitation, **c** Farmland Temperature

Table S4. Causation inference results for farmland NPP and influencing climate factors

Method	Relationship	Precipitation		Temperature	
		r/*ρ*/*b*	*p*/*ste*	*r*/*ρ*/*b*	*p*/*ste*
Pearson Correlation		0.76	0.00**	0.66	0.00**
GCCM	Climates xmap NPP	0.00 (-0.08,0.08)	0.50	0.18 (0.1,0.26)	0.00**
	NPP xmap Climates	0.48 (0.42,0.54)	0.00**	0.50 (0.43,0.56)	0.00**
LiNGAM	NPP → Climates	0.00	1917.59	0.00	30.19
	Climates → NPP	0.00	1373.85	0.00	1286.96

**** correlation is significant at the 0.01 level, * correlation is significant at the 0.05 level.**
***r*/*ρ*/*b* *r* is for Pearson correlation, *ρ* is for GCCM, *b* is for LiNGAM**
***p*/*ste* *p* is is significance *p*-value for Pearson correlation and GCCM, *stde* is the standard deviations of the estimated residuals of LiNGAM**
xmap means cross-mapping prediction, Climates means climate factors,→means cause
(*,*) is the 95% confidence interval

Section S5: Synthetic data cases

To investigate the robustness of GCCM with processing noise, we added different levels of noise to the heavy metal data in the first case as Equation (S1), and run the GCCM on the synthetic data.

$$S_{HM} = HM + a * rand * HM \quad \text{(S1)}$$

where HM is the original heavy metal concentration, i.e. Cu, Mg, Cd and Pb, S_{HM} is corresponding synthetic heavy metal concentration, $rand$ is a random number with in (0,1), a is a adjusting coefficient with value as 10%, 30%, 60% and 90% separately. The output of GCCM on the synthetic Cu, Mg, Cd and Pb are displayed in Fig.S5, S6 and S7 respectively. With increase of the noise, prediction skill ρ of heavy metal xmap industry pollutant density and heavy metal xmap nightlight (industry→heavy metal and residence→heavy metal) decreased. When a reached 60%, GCCM started to fail to identify some causations between heavy metal concentrations and industry (residence). When a reaches 90%, GCCM failed to identify the causations in most cases.

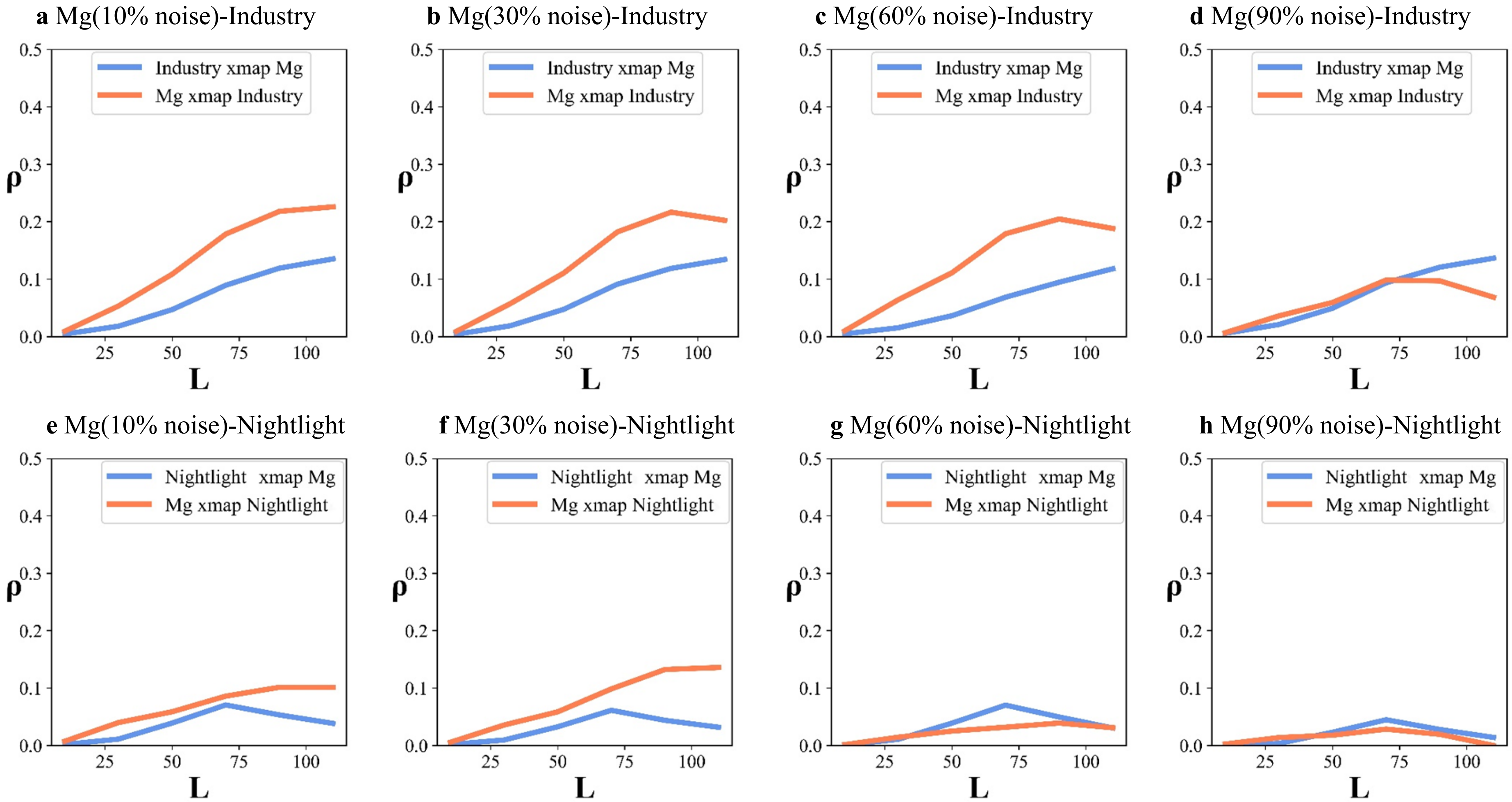

Fig.S5 Causation inference for synthetic Mg **a, b, c and d** are the cross prediction results between the industry pollution density and synthetic Mg with 10% to 30%, 60% and 90% random noise added; **e, f, g and h** are the cross prediction results between the residence and synthetic Mg with 10% to 30% , 60% and 90% random noise added.

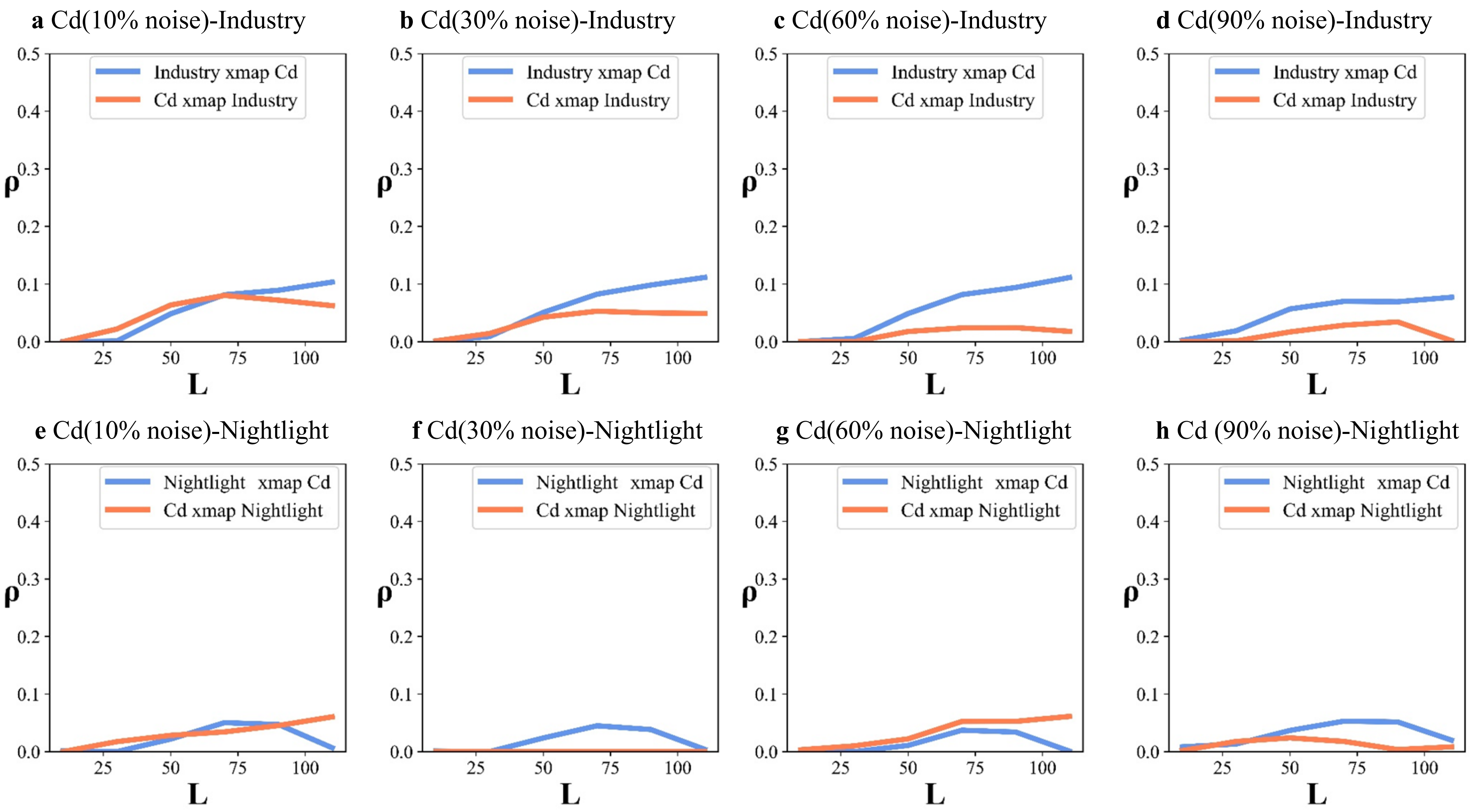

Fig.S6 Causation inference for synthetic Cd **a, b, c and d** are the cross prediction results between the industry pollution density and synthetic Cd with 10% to 30% , 60% and 90% random noise added, **e, f, g and h** are the cross prediction results between the residence and synthetic Mg with 10% to 30% , 60% and 90% random noise added.

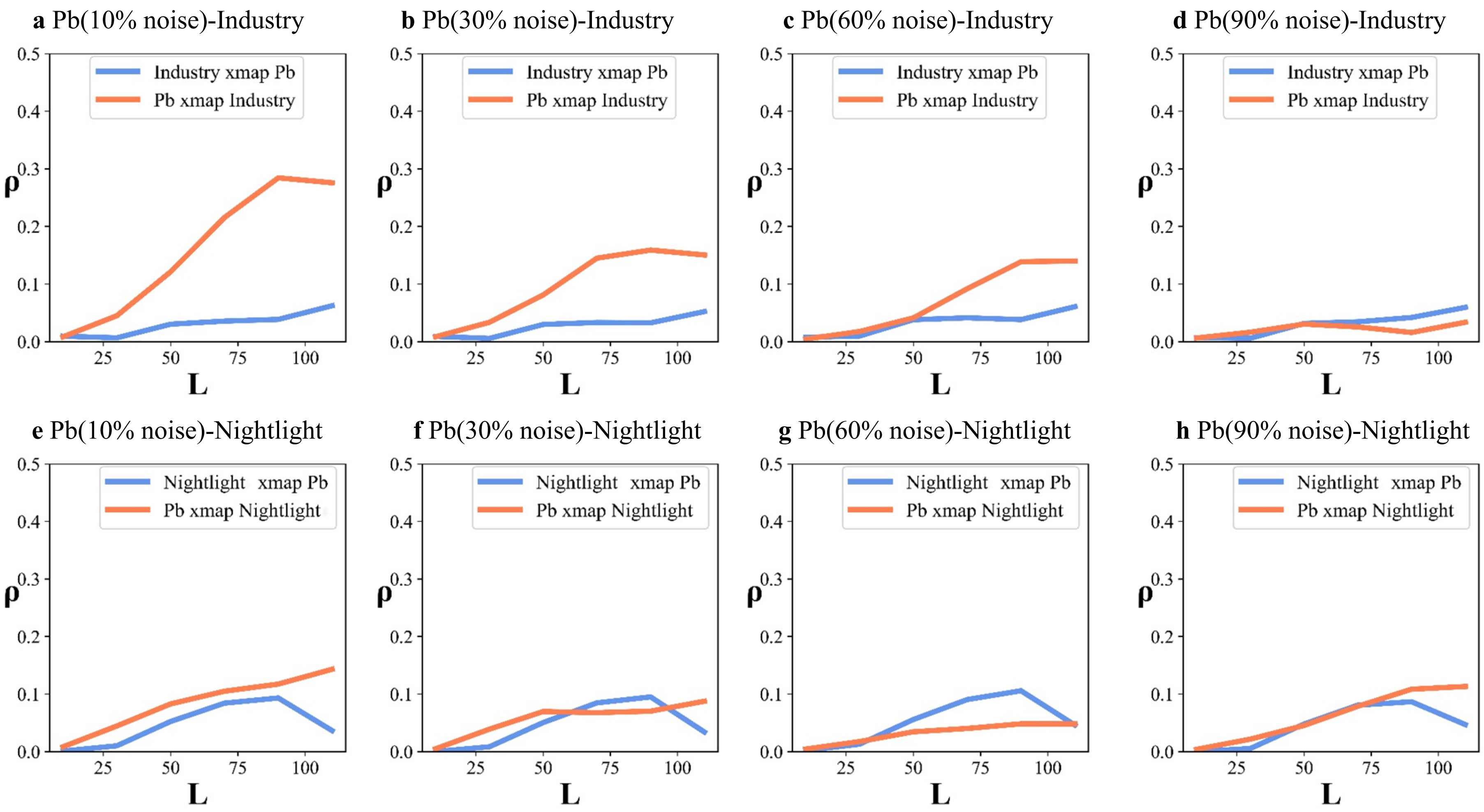

Fig.S7 Causation inference for synthetic Pb **a, b, c and d** are the cross prediction results between the industry pollution density and synthetic Pb with 10% to 30% , 60% and 90% random noise added, **e, f, g and h** are the cross prediction results between the residence and synthetic Pb with 10% to 30% , 60% and 90% random noise added.

Table S5. Causation inference results for synthetic soil heavy metals and influencing factors

**Casual associations**	Noise ratio	Cu		Cd		Mg		Pb	
		ρ	*p*	*ρ*	*p*	*ρ*	*p*	*ρ*	*p*
Industry xmap HM	10%	0.05 (-0.03,0.13)	0.09	0.10 (0.03,0.18)	0.00**	0.14 (0.06,0.21)	0.00**	0.06 (-0.01,0.14)	0.06
	30%	0.06 (-0.02,0.14)	0.07	0.11 (0.03,0.19)	0.00**	0.13 (0.06,0.21)	0.00**	0.05 (-0.02,0.13)	0.09
	60%	0.05 (-0.03,0.13)	0.11	0.11 (0.03,0.19)	0.00**	0.12 (0.04,0.19)	0.00**	0.06 (-0.02,0.14)	0.06
	90%	0.05 (-0.03,0.13)	0.09	0.08 (0,0.15)	0.02*	0.14 (0.06,0.21)	0.00**	0.06 (-0.02,0.14)	0.06
HM xmap Industry	10%	0.24 (0.17,0.31)	0.00**	0.06 (-0.01,0.14)	0.06	0.23 (0.15,0.3)	0.00**	0.28 (0.2,0.35)	0.00**
	30%	0.16 (0.08,0.23)	0.00**	0.05 (-0.03,0.13)	0.11	0.2 (0.13,0.28)	0.00**	0.15 (0.07,0.22)	0.00**
	60%	0.1 (0.02,0.17)	0.01*	0.02 (-0.06,0.09)	0.32	0.19 (0.11,0.26)	0.00**	0.14 (0.06,0.21)	0.00**
	90%	0.14 (0.07,0.22)	0.00**	0.00 (-0.08,0.08)	0.48	0.07 (-0.01,0.14)	0.04*	0.03 (-0.04,0.11)	0.19

Nightlight xmap HM	10%	0.01<br>(-0.07,0.08)	0.43	0.01<br>(-0.07,0.08)	0.43	0.04<br>(-0.04,0.12)	0.16	0.04<br>(-0.04,0.11)	0.18
	30%	0.01<br>(-0.07,0.09)	0.39	0.00<br>(-0.07,0.08)	0.46	0.03<br>(-0.05,0.11)	0.21	0.03<br>(-0.04,0.11)	0.19
	60%	0.00<br>(-0.08,0.08)	0.49	0.00<br>(-0.08,0.08)	0.49	0.03<br>(-0.05,0.11)	0.22	0.05<br>(-0.03,0.12)	0.12
	90%	0.01<br>(-0.07,0.09)	0.42	0.02<br>(-0.06,0.1)	0.31	0.01<br>(-0.06,0.09)	0.36	0.05<br>(-0.03,0.12)	0.12
HM xmap Nightlight	10%	0.17<br>(0.09,0.24)	0.00**	0.06<br>(-0.02,0.14)	0.06	0.1<br>(0.02,0.18)	0.00**	0.14<br>(0.07,0.22)	0.10
	30%	0.16<br>(0.08,0.23)	0.00**	0.00<br>(-0.08,0.08)	0.50	0.14<br>(0.06,0.21)	0.00**	0.09<br>(0.01,0.16)	0.00**
	60%	0.07<br>(-0.01,0.14)	0.05*	0.06<br>(-0.02,0.14)	0.06	0.03<br>(-0.05,0.11)	0.21	0.05<br>(-0.03,0.12)	0.01*
	90%	0.05<br>(-0.02,0.13)	0.09	0.01<br>(-0.07,0.09)	0.42	0.00<br>(-0.08,0.08)	0.50	0.11<br>(0.04,0.19)	0.11

***p* is is significance *p*-value ,** means significant at the 0.01 level, * means significant at the 0.05 level.**

ρ is the prediction skill of GCCM

xmap means cross-mapping prediction, HM means heavy metals,→means cause

(*,*) is the 95% confidence interval

Supplementary References

1. Gao B, Wang J, Stein A, Chen Z. Causal inference in spatial statistics. *Spatial Statistics* **50**, 100621 (2022).

2. Ma H, Leng S, Chen L. Data-based prediction and causality inference of nonlinear dynamics. *Science China Mathematics* **61**, 403-420 (2018).

3. Wright S. Correlation and causation. *Journal of agricultural research* **20**, 557--585 (1921).

4. Pearl J. *Causality: Models, Reasoning and Inference*. Cambridge University Press (2000).

5. Pearl J. *Causality*. Cambridge University Press (2009).

6. Runge J. Causal network reconstruction from time series: From theoretical assumptions to practical estimation. *Chaos: An Interdisciplinary Journal of Nonlinear Science* **28**, 075310 (2018).

7. Pearl J, Mackenzie D. *The Book of Why: The New Science of Cause and Effect*. Basic Books (2018).

8. Spirtes P, Glymour C. An Algorithm for Fast Recovery of Sparse Causal Graphs. *Social Science Computer Review* **9**, 62-72 (1991).

9. Runge J*, et al.* Inferring causation from time series in Earth system sciences. *Nature Communications* **10**, 2553 (2019).

10. Runge J, Kretschmer M, Flaxman S, Sejdinovic D. Detecting and quantifying causal associations in large nonlinear time series datasets. *Science Advances* **5**, eaau4996 (2019).

11. Peters J. On the Intersection Property of Conditional Independence and its Application to Causal Discovery. *Journal of Causal Inference* **3**, 108 - 197 (2014).

12. Shimizu S, Hoyer PO, Hyvärinen A, Kerminen A. A Linear Non-Gaussian Acyclic Model for Causal Discovery. *J Mach Learn Res* **7**, 2003–2030 (2006).

13. Rubin DB. Estimating causal effects of treatments in randomized and nonrandomized studies. *Journal of Educational Psychology* **66**, 688-701 (1974).

14. Frangakis CE, Rubin DB. Principal Stratification in Causal Inference. *Biometrics* **58**, 21-29 (2002).

15. Yao L, Chu Z, Li S, Li Y, Gao J, Zhang A. A Survey on Causal Inference. *ACM Trans Knowl Discov Data* **15**, Article 74 (2021).

16. Imbens GW, Rubin DB. *Causal Inference for Statistics, Social, and Biomedical Sciences: An*

Introduction. Cambridge University Press (2015).

17. Delgado MS, Florax RJGM. Difference-in-differences techniques for spatial data: Local autocorrelation and spatial interaction. *Economics Letters* **137**, 123-126 (2015).

18. Kolak M, Anselin L. A Spatial Perspective on the Econometrics of Program Evaluation. *International Regional Science Review* **43**, 128-153 (2020).

19. Granger CWJ. Investigating Causal Relations by Econometric Models and Cross-spectral Methods. *Econometrica* **37**, 424-438 (1969).

20. Sugihara G*, et al.* Detecting Causality in Complex Ecosystems. *Science* **338**, 496-500 (2012).

21. Ma H, Aihara K, Chen L. Detecting Causality from Nonlinear Dynamics with Short-term Time Series. *Sci Rep* **4**, 7464 (2014).

22. Leng S*, et al.* Partial cross mapping eliminates indirect causal influences. *Nature Communications* **11**, 2632 (2020).

23. Hengl T, Nussbaum M, Wright MN, Heuvelink GBM, Gräler B. Random forest as a generic framework for predictive modeling of spatial and spatio-temporal variables. *PeerJ* **6**, e5518 (2018).

24. Li Y, Yao N, Chau HW. Influences of removing linear and nonlinear trends from climatic variables on temporal variations of annual reference crop evapotranspiration in Xinjiang, China. *Science of The Total Environment* **592**, 680-692 (2017).

25. Lu J, Carbone GJ, Gao P. Detrending crop yield data for spatial visualization of drought impacts in the United States, 1895–2014. *Agricultural and Forest Meteorology* **237-238**, 196-208 (2017).

26. Xu X*, et al.* China Multi-period Land Use Remote Sensing Monitoring Dataset (CNLUCC). *Data Registration and Publishing System of the Resource and Environmental Science Data Center of the Chinese Academy of Sciences*, (2018).
